# Improved quality of Kambing Kacang sexing frozen semen with the addition of green tea extract

**DOI:** 10.5455/javar.2022.i609

**Published:** 2022-09-30

**Authors:** Tri Wahyu Suprayogi, Suherni Susilowati, Tatik Hernawati, Farah Ghifara Hafidha, Citra Ayu Wening, Ahmad Budi Purnawan

**Affiliations:** 1 Department of Veterinary Reproduction, Faculty of Veterinary Medicine, Universitas Airlangga, Surabaya, Indonesia; 2 Center for Artificial Insemination, Singosari, Malang, Indonesia

**Keywords:** Sexed semen, green tea extract, cryopreservation, postthawing quality, gene pool

## Abstract

**Objective:**

The objective of this study was to determine the effect of adding various doses of green tea extract to the semen of Kacang goats during the sexing process on motility, viability, membrane integrity, malondialdehide (MDA), and deoxyribonucleic acid (DNA) fragmentation.

**Materials and Methods:**

It started with the containment of the semen of the Kacang goat, followed by macroscopic and microscopic examinations. If the semen was considered viable, a diluter that had been added with various doses of green tea extract would be added to the semen. After that, sexing was carried out using the percoll gradient density medium. Next, the sexed semen was cryopreserved in liquid nitrogen. Then, an examination of motility, viability, membrane integrity, MDA, and DNA fragmentation was conducted.

**Result:**

There was a significant difference between the control and treatment (*p* ≤ 0.05). The highest result was obtained in the treatment of adding 0.05 mg of green tea extract/100 ml of Andromed^®^.

**Conclusion:**

The addition of green tea extract can improve the quality of the sexed semen of the Kacang goat after it has been cryopreserved.

## Introduction

The Kacang goat is an endemic Indonesian goat. Its size is small and it can withstand hot temperatures. It can also eat and feed efficiently, is highly adaptive, very fertile, and can give birth twice a year [1]. As the demand for goat meat grows yearly, Indonesian breeders continue to produce Kacang goats [2]. Aside from its affordable price, goat meat is savory and tender [3].

Reproductive technologies that produce superior livestock clones include artificial insemination (AI), embryo transfer, spermatozoa separation (sexing), *in-vitro* fertilization, preservation, cryopreservation, and genetic engineering. The discovery of livestock reproduction technology can be used to address the problems faced in increasing the population, production, and productivity of livestock, in terms of both quality and quantity [4]. An effective and efficient method that can be used to increase the productivity of goats is AI. Implementing AI technology will be even more useful if the sex of the animal that will be born can be determined in accordance with the purpose of an animal farm. For example, a dairy goat farm would prefer female goats, while a meat goat farm would prefer males [5]. To achieve this goal, when the female animal is ready to mate, spermatozoa that have been separated/sexed are needed (X spermatozoa and Y spermatozoa). Spermatozoa sexing is carried out by separating the X and Y chromosomes based on differences in morphological characteristics, deoxyribonucleic acid (DNA) content, differences in macromolecular proteins on the two chromosomes, and differences in weight and movement of spermatozoa [6]. To ensure that sexed spermatozoa can be stored in the long term, the spermatozoa must be cryopreserved. In the cryopreservation process, it is necessary to begin with the dilution of the cement to prevent cold shock, followed by the addition of glycerol, which serves as an intracellular cryoprotectant that protects the spermatozoa during cryopreservation and thawing.

The main obstacle to the cryopreservation process is the occurrence of cell damage, which can happen if the dehydration process does not occur, which in turn will result in the formation of intracellular ice crystals that may damage the cells. In addition, the increase in the osmolarity of the cryopreservation medium caused cell damage [7]. Susceptibility to cold temperatures is associated with a higher ratio of unsaturated fatty acids compared to saturated fat. In such a case, the number of reactive oxygen species (ROS) formed will also increase, ultimately generating malondialdehyde [8]. ROS formed decreases the motility, viability, and integrity of the plasma membrane of spermatozoa. These instances can be minimized by adding antioxidants to the diluter [9]. One of the antioxidants used to prevent ROS in spermatozoa is green tea extract [10]. Green tea extract has two components acting as antioxidants [11]: polyphenols and flavonoids [12]. Polyphenols are nonenzymatic antioxidants that prevent chain reactions [9,13], while flavonoids are antioxidants that can repair cells damaged by free radicals. Both components synergize to maintain cell life. Green tea extract contains polyphenols like catechin, epicatechin, epigallocatechin, epicatechin gallate, and epigallocatechin 3 gallate. It is reported to have a more potent antioxidant effect than vitamin C or E [14]. Therefore, the researchers wanted to find out how well green tea extract works to keep sexed goat sperm from freezing.

## Materials and Methods

### Ethical approval

This study did not need ethical clearance as the experimented animals were not tortured or killed. However, research ethics followed the guidelines set by the university’s ethics committee.

### Research period and location

This study was conducted from April to November 2020. The containment, sexing, and cryopreservation of semen were conducted at BBIB (Center for Artificial Insemination), Singosari, Malang. Motility, viability, membrane integrity, and DNA fragmentation were examined at the Cryopreserved Semen Laboratory of FKH (Faculty of Veterinary Medicine), Unair, Gresik. On the contrary, malondialdehide (MDA) levels were examined at the Biochemistry Laboratory of FKH Unair, Surabaya.

### Green tea extract

Green tea leaves were dried and ground to a particle size of 0.75 µm in a grinding machine. Green tea powder was soaked by maceration using 96% ethanol solvent, allowed to stand for 3 days, and covered with aluminum foil. The soaked substance was squeezed through filter paper, evaporated at 50°C in a rotary evaporator at 45 rpm to obtain a thick extract, and then freeze-dried and stored at −20°C until required.

#### Setting up sexing medium

The medium used was the percoll density gradient medium. The X and Y sperms were separated using percoll medium by arranging 10 layers from the highest to the lowest density (65%, 60%, 55%, 50%, 45%, 40%, 35%, 30%, 25%, and 20%) and carrying out centrifugation at 2250 rpm (850G) for 5 min. The extender used was Andromed^®^, which was added with aquabidest. The ratio between Andromed^®^ and aquabidest was 1:4 [15].

#### Semen containment and spermatozoa separation

Goat semen was contained in the artificial vagina and examined. Macroscopic examination included volume, color, odor, consistency, and pH. Microscopic examination included mass movement, individual movement, viability, concentration, and resistance. The viable semen sample was washed by centrifugation (150 × 10^6^/ml concentration), put in a separation tube, and left for 20 min. Each semen fraction was suctioned, washed, and evaluated.

#### Semen evaluation

A spermatozoa sample was prepared. Then, the length and width of the spermatozoa head were measured under a microscope with 10 × 100 magnification using a micrometer. Alternatively, eosin-nigrosin staining can be conducted. Spermatozoa that fully reflected fluorescent light were predicted to carry the X chromosome. On the contrary, those that partially reflected or did not reflect fluorescent light were expected to have the Y chromosome [14].

#### Addition of green tea extract to the diluter

The amount of diluter that would be added to each semen was calculated using the following formula:


$$\mathrm{Diluter}\;\mathrm{volume}\;=\;\frac{\mathrm{Semen}\;\mathrm{volume}\;\times \;\mathrm{Spermatozoa}\;\mathrm{concentration}\;\times \;\mathrm{Motility}}{\mathrm{The}\;\mathrm{desired}\;\mathrm{concentration}\;\mathrm{of}\;\mathrm{spermatozoa}\;\mathrm{per}\;\mathrm{milliliter}}$$

In this study, green tea was added after the mixing process of the semen with the Andromeda diluter. The concentrations added were 0.05 mg, 0.1 mg, and 0.15 mg/100 ml of diluter. Next, cryopreservation was performed at a cold temperature.

#### Cryopreserved semen evaluation

After the semen had been cryopreserved, it was thawed by immersing straws containing the semen in warm water at 37°C for 30 sec. Then, the motility, viability, membrane integrity, MDA levels, and DNA fragmentation were examined.

### Protocol of semen evaluation

#### Assessment of sperm quality

Six straws of semen from each group were used in this study. They were thawed in sterile water at 37°C for 30 sec (six replications on each parameter).

**Sperm viability:** postthawed semen was dripped on object glass, eosin-nigrosin was added and mixed homogeneously, and the mixture was smeared and dried over a flame. One hundred spermatozoa were examined under a light microscope (Olympus BX-53, Shinjuku City, Tokyo, Japan) at 400× magnification. The heads of live spermatozoa were transparent, whereas dead spermatozoa showed damage to the plasma membrane. Permeability increased gradually as the dye entered the cell, and the head appeared reddish [14].

**Sperm motility:** A wet mount was made using a 10 µl drop of semen placed directly on a microscope slide and covered with cover glass [16]. The progressive movement of spermatozoa was observed under the light microscope at 400× magnification. Sperm motility was counted on three microscopic fields for each semen sample and then averaged [14].

**Intact plasma membranes:** The hypoosmotic swelling (HOS) test was used to evaluate intact plasma membranes [17]. One ml of hypoosmotic solution (7.35 gm of sodium citrate, 13.52 gm of fructose dissolved in 1,000 ml of aqua dest) was added to 0.1 ml of spermatozoa, then incubated at room temperature for 30 min. Following incubation, 15 µl of the sample was placed on an object-glass slide, covered with a cover glass, and observed using a 400x magnification microscope (Olympus BX-53). The damaged plasma membranes were characterized by straight tails [18]. However, the circular tails marked the intact plasma membrane.

**MDA concentration:** The thiobarbituric acid method was used for MDA concentration measurement.

**DNA fragmentation:** Acridine orange staining was used to evaluate DNA fragmentation. One drop of semen dripped on the glass object, mixed with acridine orange, and covered under a fluorescent microscope at 100x magnification per 100 spermatozoa. Spermatozoa marked by DNA fragmentation are yellow to red while living ones are greenish [19].

### Data analysis

The data obtained were analyzed using the analysis of variance (ANOVA) test. If there were any differences, the Duncan test would be performed [20]. The sperm quality was evaluated based on its motility, viability, IPM, MDA concentration, and DNA fragmentation. Sperm quality among the group was assessed with ANOVA, followed by the Tukey Honestly Significant Difference test. Statistical Product and Service Solutions version 23 was used to conduct all of the statistical analysis to a 95% level of significance.

## Results and Discussion

The results of this study can be seen in the Appendix . The results were tables showed the results of macroscopic and microscopic examination of the semen of Kacang goat before treatment; the results of the study on the variable of spermatozoa motility percentage; the results of the study for the variable of viability percentage; and the variable of the membrane integrity percentage of Kacang goats’ sexed semen that has been cryopreserved; and the highest average of malondialdehyde levels.

Table 1 shows the results of macroscopic and microscopic examination of the semen of the Kacang goats before treatment. The quality was considered very good. Semen quality is generally influenced by several factors, including feed [20], breed, genetics [22], age [22], disease, environment [23], frequency of collection, transportation, exercise, and containment method [24,25]. In general, the volume of semen will increase according to age, body size, change of condition, health of reproductive organs, and frequency of semen containment [26]. The color, consistency, and concentration of spermatozoa are closely related to each other. The waterier the semen, the lower the concentration of spermatozoa and the paler the color of the semen. Meanwhile, semen consistency depends on the ratio of spermatozoa and seminal plasma [27]. The degree of acidity of its pH greatly affects the life span of spermatozoa. If the pH is too high or too low, the spermatozoa will die. The degree of acidity of semen may be influenced by the concentration of lactic acid produced in the final process of metabolism. The metabolism of spermatozoa will accumulate lactic acid. In anaerobic conditions, it can also raise or lower the pH of the sperm [25].

Table 2 shows the results of the study on the variable of spermatozoa motility percentage. The lowest result was 11.7 ± 2.5819 in the treatment of P3X = X chromosome that was added with 0.15 mg of green tea extract/100 ml of diluter, while the highest result was 32.5 ± 2.7386 in the treatment of P1Y = Y chromosome that was added with 0.05 mg of green tea extract/100 ml of diluter.

The percentage of spermatozoa’s progressive motility is an important indicator to determine the quality of semen, as the progressive motility will allow the spermatozoa to reach the infundibulum to penetrate the ovum [28]. The highest motility of Kacang goats’ spermatozoa was found in the treatment of Y chromosome that was added with 0.05 mg of green tea extract/100 ml of diluter. That particular dose of green tea is optimal for preventing ROS formation. However, it should be noted that the semen dilution, sexing, and cryopreservation processes will cause oxidative stress on spermatozoa, which can form ROS. This is supported by other researchers who state that semen from different types of males will have different resistance to extreme processes [29].

Table 2 also shows the results of the study for the variable of the viability percentage of Kacang goats’ semen that has been cryopreserved. The lowest result was found in the P3X group (X chromosome that was added with 0.15 mg of green tea extract/100ml of diluter) of 19.1 ± 4.4831, while the highest result was found in the P1Y group (Y chromosome that was added with 0.05 mg of green tea extract/100 ml of diluter) of 54.4 ± 4.3019. The semen dilution, sexing, and cryopreservation processes will cause damage to the membrane structure, especially in the semen cryopreservation process. As a result, the concentration of intracellular electrolytes will increase and cause the formation of ice crystals. If this happens continuously, the spermatozoa will die. Cell membranes, specifically the plasma and mitochondrial membranes, could be damaged during the dilution, sexing, cooling, cryopreservation, and thawing processes. In the worst-case scenario, the nucleus can also be damaged [30]. The highest viability percentage of Kacang goats’ sexed spermatozoa was found in the treatment of the Y chromosome that was added with 0.05 mg of green tea extract/100 ml of diluter.

Table 2 shows the results of the study for the variable of the membrane integrity percentage of Kacang goats’ sexed semen that has been cryopreserved. The lowest result was found in the P3X group (X chromosome that was added with 0.15 mg of green tea extract/100ml of diluter) of 14.8 ± 3.1417, while the highest result was found in the P1Y group (Y chromosome that was added with 0.05 mg of green tea extract/100 ml of diluter) of 42.8 ± 2.6191.

**Table 1.** Macroscopic and microscopic examinations of Kacang goats’ semen before treatment.

Macroscopic examination	Microscopic examination
Volume: 1.02 ± 0.4007 ml Consistency: dense Color: white custard Odor: sting pH: 6-7	Mass movement: ++ until +++ individual movement/ Motility: 88,5/3,2 Concentrate: 3,794,1 ± 1,144,045 jt/ml Viability: 98% ± 1,34 Membrane integrity: 90% ± 2,16

**Table 2.** Mean ± SD of motility, viability, and membrane integrity examination (%) spermatozoa sexing Kacang goats’ various treatments after freezing.

Treatment	Variable		
	Motility (%)	Viability (%)	Membrane Integrity (%)
P0Y P1Y P2Y P3Y P0X P1X P2X P3X	27.5^c^ ± 2.7386 32.5^d^ ± 2.7386 22.5^b^ ± 2.7386 12.5^a^ ± 2.7386 14.2^a^ ± 2.0412 25.8^bc ^± 4.9159 13.3^a^ ± 2.5819 11.7^a^ ± 2.5819	48.3^d^ ± 5.4994 54.4^e^ ± 4.3019 42.9^c^ ± 1.3371 25.2^b^ ± 1.5904 23.8^ab^ ± 1.7209 41.6^c^ ± 7.3344 21.0^ab^ ± 3.4664 19.1^a^ ± 4.4831	36.9^d^ ± 4.4089 42.8^e^ ± 2.6191 32.7^c^ ± 2.4587 18.8^a^ ± 1.9381 19.1^b^ ± 1.6565 33.6^cd^ ± 5.7460 17.1^ab^ ± 2.7646 14.8^ab^ ± 3.1417

a, b, c, d, e Different notations in the same column showed significant differences (*p* <0.05).

P0Y = Y chromosome without green tea extract (Control)

P1Y = Y chromosome plus 0.05 mg/100 ml diluter green tea extract.

P2Y = Y chromosome plus 0.10 mg/100 ml diluter green tea extract.

P3Y = Y chromosome plus 0.15 mg/100 ml green tea extract diluter.

P0X = X chromosome without green tea extract (Control).

P1X = X chromosome plus 0.05 mg/100 ml green tea extract diluter.

P2X = X chromosome plus 0.10 mg/100 ml diluter green tea extract.

P3X = X chromosome plus 0.15 mg/100 ml green tea extract dilute.

**Table 3.** Mean ± SD examination of malondialdehyde levels (nmol/ml) and fragmentation DNA (%) sexing spermatozoa of Kacang goats’ various treatments after freezing.

Treatment	Variable	
	Malondialdehid levels (nmol/ml)	Fragmentation DNA (%)
P0Y P1Y P2Y P3Y P0X P1X P2X P3X	2.7145^a^ ± 0.1265 2.5432^a^ ± 0.0778 3.1728^b^ ± 0.0173 3.7652^c^ ± 0.0503 3.2170^a^ ± 0.1168 3.1193^a^ ± 0.0569 3.5533^b^ ± 0.0963 3.9668^c^ ± 0.4470	4.0^ab^ ± 0.6324 3.5^a^ ± 0,8366 5.3^c^ ± 0.8165 7.5^e^ ± 1.0488 6.1^d^ ± 0.7527 5.0^c^ ± 0.8944 6.3^d^ ± 0.8165 7.8^e^ ± 0.7527

a, b, c, d, e Different notations in the same column showed significant differences (*p* < 0.05).

P0Y = Y chromosome without green tea extract (Control).

P1Y = Y chromosome plus 0.05 mg/100 ml diluter green tea extract.

P2Y = Y chromosome plus 0.10 mg/100 ml diluter green tea extract.

P3Y = Y chromosome plus 0.15 mg/100 ml green tea extract diluter.

P0X = X chromosome without green tea extract (Control).

P1X = X chromosome plus 0.05 mg/100 ml green tea extract diluter.

P2X = X chromosome plus 0.10 mg/100 ml diluter green tea extract.

P3X = X chromosome plus 0.15 mg/100 ml green tea extract diluter.

The function of spermatozoa’s plasma membrane plays an essential role as a filter for the exchange of intra- and extracellular substances maintained in the metabolic process [23]. Damage to the cell membrane will increase the permeability of the cell membrane. In such a case, substances that should not pass through the cell membrane can freely enter and leave the cell, disrupting the spermatozoa’s membrane integrity [31].

The results of the study showed that the sexed semen of Kacang goats bearing the Y chromosome that was added with 0.05 mg of green tea extract/100 ml of diluter was the best in maintaining the membrane integrity of the spermatozoa of Kacang goats compared to other chromosomes that were not added with any or added with other doses of green tea extract.

Table 3 shows that the highest average of malondialdehyde levels (nmol/ml) was found in the P3X group (X chromosome that was added with 0.15 mg of green tea extract/100 ml of diluter) of 3.9668 ± 0.4470. The lowest was found in the P1Y group (Y chromosome that was added with 0.05 mg of green tea extract/100 ml of diluter) of 2.5432 ± 0.0778.

A high concentration of MDA indicates the occurrence of the oxidation process in cell membranes [32]. The death of spermatozoa can be caused by many factors, one of which is the formation of ROS as a result of metabolism, dilution, cooling, cryopreservation, and thawing processes. The Y chromosome of Kacang goats’ semen that was added with 0.05 mg of green tea extract/100 ml of diluter was the best at preventing the formation of ROS.

The percentage of DNA fragmentation of Kacang goat’s sexed semen that was added with green tea extract after cryopreservation can be seen in Table 3. The highest percentage of DNA fragmentation was 7.8 ± 0.7527 in the treatment of P3X (X chromosome that was added with 0.15 mg of green tea extract/100 ml of diluter), while the lowest percentage was 3.5 ± 0.8366 in the treatment of P1Y (Y chromosome added with 0.05 mg of green tea extract/100 ml of diluter).

The Y chromosome that was added with 0.05 mg of green tea extract/100 ml of diluter was the best in preventing the occurrence of DNA fragmentation on the sexed spermatozoa of Kacang goats that had been cryopreserved. DNA fragmentation can be caused by spermatogenesis, spermatozoa maturation, oxidative stress, and infection [33]. The sexing and cryopreservation processes can cause oxidative stress, resulting in spermatozoa infertility. The optimal dose to neutralize the formation of ROS caused by oxidative stress was 0.05 mg of green tea extract/100 ml of diluter. When that amount of green tea extract is added, the spermatozoa’s DNA will not break apart.

The observed variables showed that adding 0.05 mg of green tea extract/100 ml of diluter was the most optimal. On the other hand, adding green tea extract above that dose (0.10 mg/100 ml and 0.15 mg/100 ml) would cause a decrease in the quality of the sexed goats’ semen that had been cryopreserved. This decrease in quality is caused by excess antioxidants, which results in a reverse reaction. Molecules that previously inhibited the formation of free radicals will turn into molecules that induce the formation of free radicals (pro-oxidants). This would result in oxidative stress, which can harm cells [34,35].

Between the Y and X chromosomes, generally, the Y chromosome is of better quality, as evident from the addition of various doses of green tea extract. In the sexing process, the Y chromosome can pass through the percoll density gradient medium more easily as it has a smaller size. Thus, the number of free radicals or MDA formed will also become smaller. Furthermore, with the addition of green tea extract as an antioxidant, the MDA formed becomes easier to neutralize. Therefore, it can be concluded that the addition of 0.05 mg green tea extract/100 ml of diluter on the sexing medium can increase the percentage of motility, viability, and membrane integrity of spermatozoa and reduce malondialdehyde levels and the percentage of fragmentation of Kacang goats’ sexed spermatozoa that have been cryopreserved compared to the control group and different dose groups.

## Conclusion

According to the results and discussion above, it can be concluded that there is a significant effect of adding various doses of green tea extract to the semen of Kacang goats during the sexing process on motility, viability, membrane integrity, MDA, and DNA fragmentation. The addition of green tea extract can improve the quality of the sexed semen of the Kacang goat after it has been cryopreserved. The researchers hoped that further research into another material would enhance the quality of the sexed semen of Kacang goats. Moreover, this paper could be a reference for other research.

## List of abbreviations

AI, Artificial insemination; MDA, Malondialdehyde; DNA, Deoxyribonucleic acid; ROS, Reactive oxygen species; IPM, Intact plasma membrane; ANOVA, Analysis of variance.
